# Effect of Fortification with Raspberry Juice on the Antioxidant and Potentially Anti-Inflammatory Activity of Wafers Subjected to In Vitro Digestion

**DOI:** 10.3390/foods10040791

**Published:** 2021-04-07

**Authors:** Urszula Szymanowska, Monika Karaś, Urszula Złotek, Anna Jakubczyk

**Affiliations:** Department of Biochemistry and Food Chemistry, University of Life Sciences, Skromna 8, 20-704 Lublin, Poland; urszula.szymanowska@up.lublin.pl (U.S.); monika.karas@up.lublin.pl (M.K.); anna.jakubczyk@up.lublin.pl (A.J.)

**Keywords:** raspberry juice, wafers, antioxidant activity, anti-inflammatory activity, consumer acceptance

## Abstract

The aim of this study was to investigate the effect of raspberry juice addition on the content of phenolic compounds and the antioxidant activity of wafers. The research was carried out on non-supplemented wafers (control) and wafers in which water was replaced with raspberry juice in the amount of 10%, 20%, 50%, 75% and 100%. The potential bioavailability of the phenolic compounds after in vitro digestion was also determined. As shown by the consumer assessment, wafers in which the water was replaced with 100% raspberry juice turned out to be the best variant of the enriched wafers. The content of total phenolic compounds, flavonoids, phenolic acids, and anthocyanins in the tested products increased with the increasing amount of raspberry juice added to the wafers. The fortification of the wafers with raspberry juice had a positive effect on the antioxidant activity, expressed as the ability to neutralize free radicals ABTS (2,2′-azino-bis(3-ethylbenzthiazoline-6-sulfonic acid)) and DPPH (di(phenyl)-(2,4,6-trinitrophenyl)iminoazanium), the ability to chelate iron ions, and as the reduction power. The simulated digestion increased the content of phenolic compounds and increased the antioxidant activity of the wafers. The ability to inhibit lipoxygenase and cyclooxygenase 1 and 2 (i.e., enzymes involved in the induction of inflammation), varied and depended on both the amount of raspberry juice added and the type of extract.

## 1. Introduction

Oxidative stress occurs when the generation of reactive oxygen species (ROS) in an organism’s cells exceeds the ability of the system to neutralize and eliminate them. In recent years, many scientific studies have shown that oxidative stress in the human organism has been implicated in different health problems, including neurological, cardiovascular, and inflammatory diseases, as well as cancer [1,2]. The excess of free radicals leading to oxidative stress is neutralized by exogenous food-derived antioxidants [3,4]. The antioxidant potential of food products, especially vegetables and fruits, is considered to be an indicator of their pro-health effects [5]. Antioxidants play a protective role in the pathogenesis of oxygen-mediated diseases [6]. Inflammation is closely related to the occurrence of oxidative stress in the cell. In addition to damage to proteins and DNA and lipid peroxidation, the overproduction of free radicals induces the production of pro-inflammatory cytokines and leads to chronic inflammation. Arachidonic-acid-metabolizing enzymes (i.e., lipoxygenase and cyclooxygenase) are involved in the development of inflammation. Phenolic compounds, especially flavonoids and anthocyanins, can reduce inflammation by acting as inhibitors of these enzymes [4,7,8]. Raspberry fruits are especially effective, as their high antioxidant potential supports the natural systems of cell protection against the harmful effects of free radicals [9,10].

Raspberry fruits are a rich source of phenolic compounds with antioxidant properties [11,12]. Raspberry polyphenols can be divided into phenolic acids, flavonoids, and anthocyanins. Phenolic compounds are secondary plant metabolites with different molecular weight, structure, and chemical, biological, and physical properties [13]. In addition to their beneficial biological functions, phenolic compounds are closely related to sensory characteristics such as color, taste, and aroma [14].

There are two important groups of polyphenols in berries (e.g., raspberries). One is the flavonoids (especially anthocyanins and flavonols). The other group is the phenolic acids, with ellagic acid as the most important antioxidant [15,16]. Poland is the leading producer of raspberries in the world and in Europe (FAO, 2014). Due to the seasonality of harvesting and the short shelf life of these fruits, there is a need for quick processing thereof. A large proportion of the fruit is processed into juices, both in industrial and home settings [17,18]. Fruit juices have long been an important year-round component of the human diet. Their popularity is associated with not only their taste, low calorific value, or the presence of easily digestible simple sugars and mineral salts, but also with the presence of biologically active ingredients such as vitamins C and E and polyphenolic compounds [19].

Currently, food fortification with biologically active substances is becoming increasingly popular. Its aim is to increase the health quality of foods and to make the resulting products “functional foods”. The growing public interest in healthy nutrition forces food producers to modify standard products in order to enhance their attractiveness and health-promoting properties [20,21]. Plants rich in bioactive ingredients are particularly interesting additives to food products. This is mainly related to their natural origin and use in traditional medicine. Fruit juices have high nutritional and dietary values, and support the regulation of metabolism. It has been shown that phenolic compounds from juices are very well absorbed [22]. Confectionery products—especially shortbread cookies or wafers—are a good target for enrichment through the incorporation of various nutritional or bioactive ingredients [23,24,25].

Therefore, the aim of this study was to investigate the antioxidant and anti-inflammatory potential of wafers enriched with raspberry juice. Particular attention was paid to the effectiveness of fortifications in view of the potential bioavailability of phenolic compounds. 

## 2. Materials and Methods

### 2.1. Material

The research material was wheat wafers enriched with raspberry juice. The raspberry fruit (*Rubus idaeus* L. var. Polana) were purchased from a local farm in the Lublin region (Poland). The other wafer cake ingredients (i.e., wheat flour (type 500), sugar, eggs, baking powder, and butter) were purchased at a local market in Lublin, Poland. 

### 2.2. Preparation of Raspberry Juice 

Laboratory-scale raspberry juice production: Raspberry fruits (1 kg) were squeezed (Eujuicers.com 8004 squeezer, Omega, Prague, Czech Republic). The juice samples were centrifuged for 15 min at 4 °C and 9000× *g*. The volume of juice was measured and the juice was then frozen. Thawed juice was used as a substitute for water in the specified amounts to bake wafers.

### 2.3. Preparation of Wafers Enriched with Raspberry Juice 

Basic recipe for wafers: 167 g of butter (melted and cooled), 120 g of sugar, 4 eggs, 280 g of flour, 3 g of baking powder, and 240 mL of water. The wafers were prepared as the variants presented in Table 1. 

The ingredients were carefully measured. The flour, sugar, baking powder, eggs, and butter were mixed in a dish with a hand blender (Braun, GmbH, Kronberg im Taunus, Germany). The homogeneous mass was divided into six bowls and, depending on the variant, the specified amount of raspberry juice and/or lukewarm water was added. Dough portions (3 tablespoons, ~45 mL) were applied to the center of a waffle maker Clatronic HA 3494 (Kempen, Germany) and baked for approx. 1.5 min at 180 °C. After cooling, the wafers were hermetically packed and used for further analysis.

### 2.4. Extract Preparation

Ethanolic extract (E): 15 mL of 50% ethanol with 0.1% HCl was added to 2 g of each wafer variant and the mixture was homogenized for 2 min. The samples were shaken for 30 min under refrigeration and centrifuged for 15 min (9000× *g*, 4 °C) and the supernatant was decanted. The extraction was performed in triplicate, and the three fractions obtained were combined and made up to 50 mL with the solvent used.

Buffer extract (B): 15 mL of phosphate-buffered saline (PBS) was added to 2 g of each wafer variant, and the mixture was homogenized for 2 min. The samples were shaken for 30 min under refrigeration, centrifuged for 15 min (9000× *g*, 4 °C), and filtered. The extraction was performed in triplicate and the three fractions obtained were pooled and made up to 50 mL with the PBS solution.

In vitro digestion (D) was carried out according to the procedure described by Minekus et al. (2014) [26]. 

### 2.5. Polyphenol Compound Content

#### 2.5.1. Total Phenolic Content (TPC) 

The concentration of total phenolic compounds was determined using Folin–Ciocalteu reagent and expressed in mg/g as gallic acid equivalent [27].

#### 2.5.2. Flavonoid Content (FC)

The flavonoid concentrations were determined with the AlCl_3_ method and expressed in mg/g as quercetin equivalent [28].

#### 2.5.3. Phenolic Acid Content (PAC)

The phenolic acid concentrations were determined using the Arnov reagent method and expressed in μg/g as caffeic acid equivalent [29].

#### 2.5.4. Total Anthocyanin Content (TAC) 

The total monomeric anthocyanin content was determined using the pH differential method and calculated in mg/g as cyanidin-3-*O*-glucoside equivalent [30]. Before anthocyanin content measurements, the samples obtained after the in vitro digestion (D) were passed through Supelco C-18 cartridges (Sigma-Aldrich, Poznań, Poland) according to the procedure described earlier [31] to eliminate the effect of bile salts. 

### 2.6. Antioxidant Activity

#### 2.6.1. Antiradical Activity

The radical scavenging ability was determined using 2,2′-azino-bis(3-ethylbenzthiazoline-6-sulfonic acid (ABTS^•+^) and di(phenyl)-(2,4,6-trinitrophenyl)iminoazanium (DPPH^•^) according to the methods proposed by Re et al. [32] and Brand-Williams et al. [33], respectively. The results were expressed as Trolox equivalent (µM TE/g).

#### 2.6.2. Chelating Power (Fe^2+^) and Reduction Power (RP)

The ability to chelate iron(II) ions was measured based on the method used by Guo et al. [34] and expressed as EDTA equivalent (mg EDTA/g). RP was measured with the method described by Pulido et al. [35] and expressed as Trolox equivalent (µM TE/g).

### 2.7. Potential Anti-Inflammatory Activity

The inhibitory potential against lipoxygenase (LOX) was determined based on the method described by Szymanowska et al. [31]. An extract concentration (mg FW/mL) providing 50% inhibition (IC_50_) was obtained by plotting the inhibition percentage against sample concentrations. The ability to inhibit cyclooxygenase 1 and 2 (COX1 and COX2) was determined with the use of the Cayman Chemical COX Colorimetric Inhibitor Screening Assay Kit. The results are expressed as IC_50_—the concentration (mg/mL) that causes 50% inhibition.

### 2.8. Consumer Acceptance

The organoleptic evaluation of the wafer variants using a 5-point method was carried out by a research team consisting of 25 students and employees of the University of Life Sciences in Lublin. The distinguishing features taken into account by the evaluators included the shape, color, surface, texture, taste, and aroma. The evaluators entered their scores in appropriate tables on the reporting form. The quality of the individual parameters of wafers enriched with raspberry juice was assessed using a five-point hedonic scale (1: bad; 5: very good).

### 2.9. Color Determination

The instrumental color measurement was performed with the CIELab method using an EnviSense NH310 colorimeter (EnviSense, Lublin, Poland). The chroma (*C*):

*C* = (a*^2^ + b*^2^)^1/2^ (1)

and hue angle (*h*°) were also calculated:

*h*° = arctan (b*/a*) (2)

The total color differences (Δ*E*) between the non-fortified wafers and those with the raspberry juice additive were calculated using the following formula:

(3) $$\Delta E=\sqrt{{\left(\Delta L^{*}\right)}^{2}+{\left(\Delta a^{*}\right)}^{2}+{\left(\Delta b^{*}\right)}^{2}}$$

where L* (lightness), a* (redness), and b* (yellowness) and ΔL*, Δa*, and Δb* are differences between the control and fortified wafers [36].

### 2.10. Statistical Analysis 

The experimental data are shown as mean ± S.D. of data obtained from three independent samples of each extract in three parallel experiments (*n* = 9). Statistical analysis was carried out using STATISTICA 13.1 for mean comparison using Tukey’s test at the significance level *p* ≤ 0.05.

## 3. Results

### 3.1. Sensory and Color Evaluation

**Figure 1:**
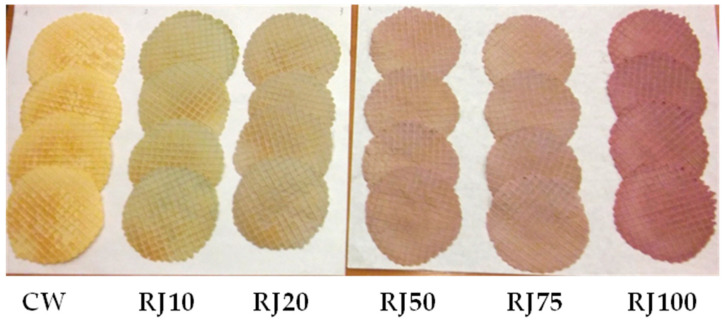
Control wafers (CW) and wafers fortified with raspberry juice (RJ10–RJ100, from 10% to 100% water substitution with raspberry juice, respectively).

Wafers enriched with raspberry juice are shown in Figure 1. 

**Figure 2:**
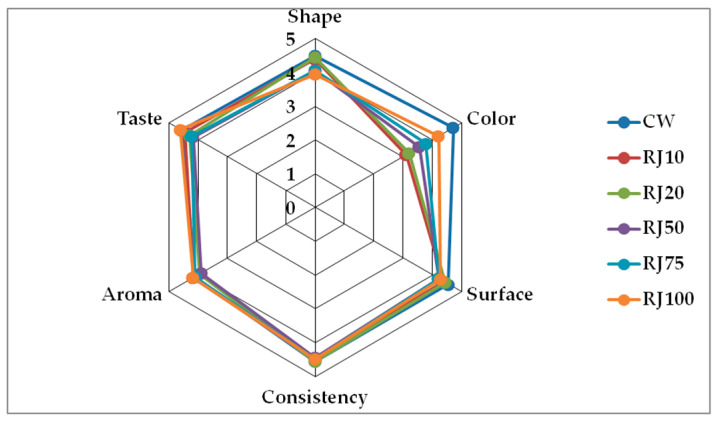
Sensory properties of the analyzed wafers.

Freshly baked and cooled wafers were subjected to organoleptic evaluation. The results of the evaluation of the quality factors are presented in Table 2 and Figure 2. 

The shape of the wafers received scores from 3.94 ± 0.25 for the RJ100 variant to 4.5 ± 0.29 for the CW. This parameter was likely influenced by the viscosity of the juice, which caused visual changes in the shape of the product. However, these differences were statistically insignificant. In terms of the color of the wafers, the RJ10 wafers scored the lowest (3.11 ± 0.16) and the control wafers the highest (4.72 ± 0.25). It is worth noting that the RJ100 variant was only slightly worse than the control wafers (4.22 ± 0.22). The color of the fortified wafers was related to the presence of anthocyanin dyes in the raspberry juice. The higher the addition of raspberry juice to the wafer dough (i.e., the higher the concentration of anthocyanins), the more noticeable the color change. However, the wafers with added juice were only attractive to consumers at replacement levels above 50%. Consumers are not used to changes in the color of wafers. Wafers with a small amount of juice had an unappetizing greenish color. There were slight differences in the assessment of the surface, probably influenced by the juice viscosity. The consistency of the wafers was generally highly rated (over 4.4), which proves their high quality. The smell of the wafers with toasted butter aromas was rated the lowest. In the RJ100 variant, this was masked by the fruity aroma of the juice. The taste of all wafers was rated relatively high (scores above 4.2). Wafers in which 100% of the water was replaced with juice were scored highest for taste. They were sweet with a noticeable fruity, raspberry flavor. According to the evaluation team, taking into account the overall rating, the control wafer turned out to be the best option, while the rating of the RJ100 wafers was only slightly lower. It is worth noting that the differences between the individual wafer variants were generally not statistically significant. Consumers are used to the traditional taste and appearance of wafers, but they also accept new products.

The color parameters of the control and fortified wafers were also assessed with the instrumental method in the CIELab system. The results are presented in Table 3. With the increasing addition of juice, the value of the L parameter decreased, which evidences the decreasing brightness of the color. The a parameter slightly increased with the increasing concentration of raspberry juice in the wafers, which indicates a greater share of the red color. Significant differences were noted for the RJ50, RJ75, and RJ100 variants compared to the control. The b parameter had a significantly lower value as the content of raspberry juice in the wafers increased, which means that the yellow color decreased.

The chroma value (C*) is used to indicate the degree of saturation of a color, and is proportional to its strength. The higher the chroma values, the higher the color intensity of samples perceived by humans. The highest C* value was calculated for the CW wafers. The C* value for all juice-fortified wafers was significantly lower than in the control. On the hue circle, *h* = 0° denotes redness, *h* = 90° denotes yellowness, *h* = 180° denotes greenness, and *h* = 270° denotes blueness. The hue angle (*h°*) decreased with increasing raspberry juice addition in the wafers. This means that the shade of the color changed from yellowish to redder. Even a 20% addition of juice caused a significant change in the hue angle compared to the control. The lowest value (i.e., the closest to red) was noted for the RJ100 variant. Δ*E* ≥ 3 indicates that the color is perceivable by humans [37]. The results of the Δ*E* value of the wafers with the addition of raspberry juice ranged from 9 to 37, which means that the color differences between the control wafers and the enriched wafers were noticeable and statistically significant, and increased with the addition of juice. There was no statistically significant difference between RJ50 and RJ75. 

### 3.2. Phenolic Content in Wafers

**Figure 3:**
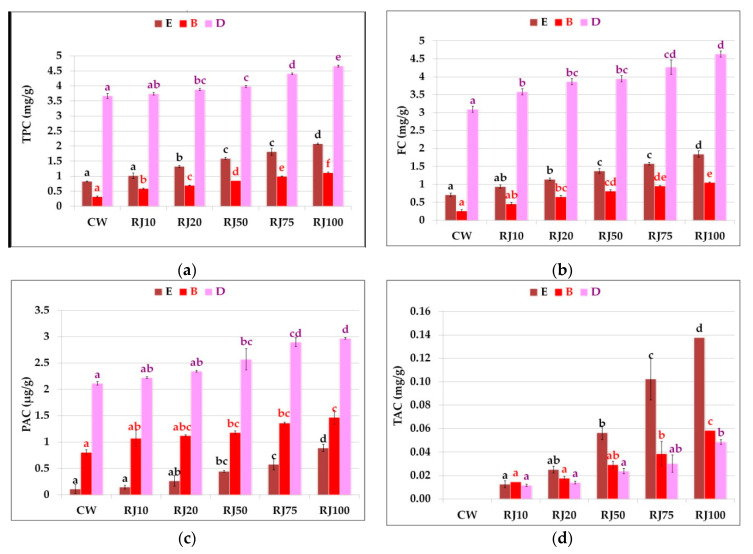
Phenolic compound content in the different extracts from the control and fortified wafers. (**a**) Total phenolic content; (**b**) flavonoid content; (**c**) phenolic acid content; (**d**) anthocyanin content. E—ethanolic extract; B—buffer extract; D—extract after in vitro digestion. CW—control wafers, RJ10–RJ100—wafers with raspberry juice addition (from 10% to 100% water substitution, respectively). All values are mean ± standard deviation for triplicate experiments. Different letters indicates statistically significant differences between the samples within the same type of extract (*p* < 0.05).

The content of total phenolic compounds and groups of polyphenols in extracts prepared from the control and fortified wafers is shown in Figure 3a–d. In all ethanol extracts (E) from wafers with the addition of raspberry juice, the total phenolic compound content was higher than in the control sample. The content of phenolic compounds increased proportionally with the increase in the raspberry juice addition (Figure 3a); however, the difference was not statistically significant in the case of the RJ10 sample. The highest amount of the tested compounds was determined in the RJ100 sample. 

The content of total phenolic compounds was higher in all PBS extracts (B) from wafers with the addition of raspberry juice than in the control sample. It was between 0.32 mg/g in the control sample and 1.11 mg/g in the RJ100 sample. The content of these compounds increased in direct proportion to the juice concentration in the tested wafers (Figure 3a). 

The content of total phenolic compounds after in vitro digestion also increased with the increase in the amount of raspberry juice in the wafers. A statistically significant increase vs. the control was observed in the RJ20 samples (Figure 3a). The comparison of the total phenolic compound content between the three extracts from the tested wafers (E, B, and D) showed significant differences. The content of phenolic compounds in the PBS extracts was lower than in the ethanol extracts, which can be explained by the fact that ethanol is a much better solvent for phenolic compounds. In turn, extracts subjected to simulated digestion had significantly higher values than the ethanol and PBS extracts.

The extraction method may affect the content of phenolic compounds and their antioxidant activity. For this reason, we tested three types of extracts: ethanolic (E) (i.e., chemical), buffer (B), and simulated digestion (D). Chemical extraction is most often used to determine the antioxidant potential. However, it does not reflect the situation in the body. In turn, extraction with buffer is mainly used for the isolation of hydrophilic antioxidants, which may lead to the underestimation of the activity of the tested samples. It is most realistic for consumers to determine the antioxidant potential of samples undergoing simulated digestion. This allows determination of the content of potentially bioavailable compounds. Our previous screening studies (unpublished data) indicated that an increase in the extraction time (for extracts E and B) had a lesser impact on the yield than the shorter but triple extraction.

In the ethanol extracts, a gradual increase in the content of flavonoids was observed with the increase in the addition of raspberry juice (Figure 3b). The amount of flavonoids was over 2.5 times higher in the RJ100 sample than in the CW sample. The content of flavonoids in the buffer extracts was lower than in the ethanol extracts, but it also increased with the increasing amount of raspberry juice. The highest content of these compounds was determined after simulated digestion. The greatest differences between the types of extracts (E, B, and D) were noted for the control sample.

The lowest levels of phenolic acids (PAC) were determined in the ethanol extracts. The content of phenolic acids in the PBS extracts was twice as high. The extracts subjected to simulated digestion had the highest content of phenolic acids. Their concentration was four times higher than in the ethanol extracts (Figure 3c). In the ethanol extracts, the content of phenolic acids in the RJ100 variant was over eight times higher than in CW. In all types of extracts, statistically significant differences with respect to the control were noted for the RJ50–RJ100 samples.

In the ethanol (E) and buffer (B) extracts, the content of anthocyanins in the wafers increased with the addition of raspberry juice. The highest concentration of anthocyanins was determined in the RJ100 sample (i.e., 0.13 mg/g and 0.06 mg/g, respectively). No anthocyanins were recorded in the control sample (Figure 3d). In the samples obtained after the simulated wafer digestion, the anthocyanin content was the lowest and ranged from 0.012 mg/g to 0.05 mg/g for the RJ10 to RJ100 samples, respectively. Anthocyanins are the most labile group of polyphenols.

### 3.3. Antioxidant Properties

The antioxidant properties of the wafers were tested using four complementary methods: the ability to scavenge free radicals ABTS and DPPH, the ability to chelate iron(II) ions, and the reduction power. The results are presented in the Table 4.

The free radical scavenging capacity determined against the ABTS cation was relatively low for extracts E and B, but increased with the addition of raspberry juice to the wafers. The extracts obtained from wafers subjected to in vitro digestion showed significantly higher anti-radical activity than the ethanolic and buffer samples.

Ethanol extracts from wafers with the addition of raspberry juice showed a statistically significantly higher ability to neutralize DPPH free radicals than the control sample. An exception was the RJ10 sample, containing a small amount of raspberry juice. No significant differences were found between samples RJ20–RJ100. The buffer extracts exhibited the lowest ability to neutralize DPPH radicals of all the wafer extracts. Simulated gastrointestinal digestion increased the scavenging capacity of DPPH radicals compared to the PBS extracts. However, these results were lower than those obtained for the alcohol extracts. This tendency may be related to the fact that this method is based on the use of an alcoholic DPPH solution in the analysis of buffer extracts.

An increased ability to chelate iron ions Fe^2+^ was observed in the ethanol extracts from wafers with the addition of raspberry juice compared to the control. In the ethanol extracts, an increase in the amount of raspberry juice added resulted in a proportional increase in the ability to chelate iron(II) ions. The RJ100 sample was characterized by the highest activity (0.36 ± 0.00 mg EDTA equivalent/g), while the activity for the CW sample was only 0.26 ± 0.01 mg EDTA equivalent/g. A similar trend was also observed for the buffer extracts and after simulated digestion. However, it should be noted that the highest chelating activity was shown by the B extracts.

The reduction power (RP) in all three types of extracts increased proportionally with the increasing amount of raspberry juice in the wafers. All recorded differences were statistically significant. The lowest results were found for the buffer extracts, whereas they were 2–3 times higher for the ethanol extracts. In turn, the highest RP (i.e., from 10.31 ± 0.37 to 24.79 ± 0.57 µM TE/g) was determined in samples from wafers subjected to digestion.

### 3.4. Potential Anti-Inflammatory Activity 

The potential anti-inflammatory effects of the wafers, expressed by their ability to inhibit the activity of lipoxygenase and cyclooxygenase 1 and 2, are presented in Table 5. The lowest LOX-inhibitory activity was determined for the buffer extracts, while the highest values of the parameter were calculated for the in-vitro-digested samples. The trend was dominant in all extracts—the greater the addition of raspberry juice, the lower the IC_50_ value (i.e., the stronger the inhibition of LOX activity). The greatest degree of inhibition was determined for sample RJ100 subjected to simulated digestion: 0.39 ± 0.01 mg/mL. 

The ethanol extracts inhibited the activity of COX2 much less potently than that of COX1. A reverse relationship was observed for the buffer and digested extracts. Here, higher inhibitory activity was determined in relation to COX2. Cyclooxygenase 2, an enzyme induced by inflammation, was most strongly inhibited by the in-vitro-digested extracts. The general trend was similar to that of LOX—that is, a greater amount of raspberry juice in the wafer dough resulted in a higher inhibitory activity against both isoforms of the COX enzyme.

## 4. Discussion

Raspberry juice has been used for generations as a remedy for flu-like infections. It is attractive to consumers due to its aroma and color, but the pure juice has a very sour taste. Confectionery products, including wheat wafers, which are one of the most frequently chosen snacks, are a good matrix for enrichment with bioactive ingredients [25]. Hence, replacing some of the water with raspberry juice in the waffle dough seems to be a good way to enrich the diet with anthocyanins. To test to what extent the addition of anthocyanins to the dough affects their potential bioavailability, the wafers were subjected to simulated digestion.

An additional aspect is the attractiveness of the final product for potential consumers. Color is one of the most important quality attributes of food, and influences consumers’ choices and preferences. The increasing consumer awareness of the importance of a healthy lifestyle creates a growing demand for natural dyes rather than synthetic ones. In our study, the wafers with added raspberry juice were darker than the control product (decrease in the *L* value), and had an increased red color (increased value of parameter *a*). These results are in agreement with findings reported by other researchers. The addition of different kinds of pomace to shortbread cookies caused a decrease in their lightness. The darkest were those with 20% elderberry pomace [24]. Similarly, the addition of raspberry pomace to the dough increased the redness of cookies, which was caused by the characteristic red cyanidine-3-phosphoroside pigment. The changes in the color of the biscuits reflected the composition of anthocyanins present in the pomace [38]. In general, the addition of raw materials containing anthocyanins, fruit, juice, or pomace causes a significant change in the color of baked goods. Additionally, the color parameters are influenced by the baking process and the Maillard reaction products formed at high temperature [39,40]. 

The consumer assessment of the prepared wafers showed the best scores for the variant of fortified wafers in which water was replaced with 100% juice. The overall acceptance was only slightly lower than that of the control wafers. In general, all wafers were assessed similarly, as the differences turned out to be statistically insignificant. Prithwa et al. found that enrichment of cookies with pomegranate juice was more acceptable than enrichment with peel powder [25]. In turn, cookies enriched with raspberry and blueberry pomace and their mixture were rated higher than control cookies [38].

The analysis of the content of phenolic compounds in this study revealed that the addition of raspberry juice increased their content in the tested wafers. Similar results were also obtained by other researchers who enriched confectionery bread with juice, fruit puree, or pomace of berries. In muffins with 10% raspberry juice, a statistically significantly higher content of polyphenols was found than in the control, regardless of the time of mixing or application of freeze-drying [41]. Similarly, studies conducted by Šarić et al. showed that 30% addition of raspberry pomace to biscuits caused a significant increase in the concentration of polyphenols compared to the control [38,39]. In the present study, the addition of raspberry juice to wafers also increased the content of particular groups of polyphenols (i.e., flavonoids, phenolic acids, and anthocyanins). A proportional increase in the concentration of flavonoids and anthocyanins in relation to the amount of added juice was also noted in cookies fortified with pomegranate juice [25]. The recovery of phenolic compounds from cookies after baking depends not only on the amount of the additive, but mainly on its composition and the sensitivity of the compounds to baking parameters. This is confirmed by studies in which raspberry and cranberry pomace was incorporated into American-style muffins prepared in various baking conditions. Anthocyanins are especially thermolabile. However, the baking time is crucial. An increase in the temperature from 140 to 240 °C, and thus shortening the baking time, increased the recovery of anthocyanins by 1.6–2 times [42]. The results obtained in this study indicate that bound polyphenols that are not extracted with solvents may be released during simulated digestion. In addition, other compounds are released from the flour (e.g., peptides) can also react with Folin’s reagent, causing an overestimation of the content of phenolic compounds. Only the content of anthocyanins decreased after the in vitro digestion, but it was comparable to the concentration in the buffer extracts. Anthocyanins are labile compounds, sensitive not only to elevated temperature, but also to changes in pH. At the alkaline pH of the intestine, anthocyanins lose their intense red color. However, codigestion with some foodstuffs may alter the bioavailability of phenols and anthocyanins, as confirmed by McDougall et al. [43]. Phenol and anthocyanin binding to foodstuffs during gastric and pancreatic digestion would protect the anthocyanins. Additionally, anthocyanin losses can occur during SPE (solid phase extraction) cleaning. Anthocyanins are stable in gastric conditions, and can be partially absorbed from the stomach [43,44].

In this study, the fortified wafers showed generally higher antioxidant activity than the control wafers. The simulated digestion improved their ability to neutralize the radical cation ABTS and also improved their RP. Nakov et al. analyzed the influence of grape (*Vitis vinifera*) pomace powder on the antioxidant properties of cakes prepared with the replacement of bread wheat flour with 4%, 6%, 8%, and 10% grape pomace powder (GPP). The enrichment with 10% GPP contributed to a significant increase in antioxidant capacity as measured with the DPPH and FRAP methods [40]. The enrichment of cookies with 20% blackcurrant and elderberry pomace resulted in a significant increase in their antioxidant properties measured by the DPPH test [24]. Raspberries (both fruit and juice and pomace) may be an effective binder of aluminum, iron, and cooper ions, due to the rich content of polyphenols. These polyphenols form complexes with metal ions at neutral pH and precipitate easily through the gut barrier [45]. This is in line with the results obtained in our work. The increase in the antioxidant activity of the wafers after simulated digestion can also be explained by the release of other compounds from the matrix (e.g., peptides), showing the anti-radical potential. The Maillard reaction products formed during baking may also be responsible for the antioxidant properties of cookies or wafers [46].

The addition of raspberry juice to wafers also influences the ability to inhibit enzymes involved in the metabolism of arachidonic acid (AA)—that is, lipoxygenase and cyclooxygenase. AA-derived eicosanoids are characterized by very high biological activity, even in very small amounts, and a very broad spectrum of biological activity, but most of all they play an important role in the regulation of the inflammatory process. There are many mechanisms of the anti-inflammatory action of polyphenolic compounds, especially flavonoids. One of them is the inhibition of LOX and COX activity [47]. Złotek also found that in vitro digestion released LOX inhibitors from basil-enriched cakes [48]. In the current study, the extracts obtained after in vitro digestion were characterized by the highest inhibitory activity in relation to these enzymes. Simulated digestion released compounds with anti-inflammatory activity. The results obtained in this study confirm the literature data. 

## 5. Conclusions

Raspberry juice can be used as a functional ingredient to improve the nutritional value and enhance the health-promoting properties and organoleptic properties of wafers. The in vitro digestion process showed that the phytochemicals contained in the wafers are potentially bioavailable. From a nutritional and functional point of view, this fraction is much more important than the total amount of bioactive compounds determined in the wafers. Obviously, further research is necessary, preferably first on cellular models, which will make it possible to evaluate the degree of absorption (bioaccessibility) of compounds released from the food. Undoubtedly, the wafers in which water was completely replaced with raspberry juice were characterized by an attractive color and were positively assessed by potential consumers.

## Figures and Tables

**Table 1:** Wafer formulation.

Wafer Variant		Water (mL)	Raspberry Juice (mL)
CW-	control wafers	40	-
RJ10-	wafers with 10% juice	36	4
RJ20-	wafers with 20% juice	32	8
RJ50-	wafers with 50% juice	20	20
RJ75-	wafers with 75% juice	10	30
RJ100-	wafers with 100% juice	-	40

**Table 2:** Sensory evaluation of control and fortified wafers, assessed using a five-point hedonic scale (1: bad; 5: very good).

Quality Attribute	Weighting Factor	Variants					
		CW	RJ10	RJ20	RJ50	RJ75	RJ100
Shape	0.10	4.5 ± 0.29	4.39 ± 0.28	4.44 ± 0.28	4.06 ± 0.23	4.06 ± 0.25	3.94 ± 0.25
Color	0.15	4.72 ± 0.25 c	3.11 ± 0.16 a	3.22 ± 0.17 a	3.56 ± 0.19 ab	3.78 ± 0.20 ab	4.22 ± 0.22 bc
Surface	0.15	4.56 ± 0.28	4.39 ± 0.27	4.44 ± 0.28	4.22 ± 0.26	4.22 ± 0.26	4.28 ± 0.27
Consistency	0.20	4.56 ± 0.10	4.5 ± 0.11	4.56 ± 0.10	4.44 ± 0.09	4.5 ± 0.10	4.5 ± 0.10
Aroma	0.15	4.11 ± 0.13	4.17 ± 0.13	3.94 ± 0.13	3.89 ± 0.12	4.11 ± 0.13	4.17 ± 0.13
Taste	0.25	4.56 ± 0.21	4.44 ± 0.20	4.28 ± 020	4.17 ± 0.19	4.22 ± 0.19	4.61 ± 0.21
Overall acceptance		4.51 ± 0.32	4.2 ± 0.30	4.17 ± 0.29	4.09 ± 0.25	4.18 ± 0.30	4.35 ± 0.31

All values are mean ± standard deviation for triplicate experiments. Values in rows designated by different letters are significantly different (*p* ≤ 0.05).

**Table 3:** Assessment of wafer color parameters.

	Color Characteristic					
Sample	L*	a*	b*	C*	*h*°	Δ*E*
CW	84.95 ± 1.06 d	5.49 ± 0.08 a	29.59 ± 0.32 e	30.09 ± 0.33 e	1.39 ± 0.00 d	-
RJ10	78.37 ± 1.20 c	5.26 ± 0.26 a	21.54 ± 0.37 d	22.17 ± 0.43 d	1.33 ± 0.01 d	9.76 ± 0.13 a
RJ20	76.94 ± 0.42 c	6.83 ± 0.13 a	14.69 ± 0.46 c	16.23 ± 0.40 b	1.13 ± 0.00 c	16.85 ± 0.17 b
RJ50	72.20 ± 0.33 b	10.13 ± 0.21 b	9.54 ± 0.47 b	13.91 ± 0.47 a	0.76 ± 0.01 b	24.21 ± 0.29 c
RJ75	71.69 ± 0.76 b	10.96 ± 0.09 b	9.66 ± 0.61 b	14.66 ± 0.47 ab	0.72 ± 0.023 b	24.57 ± 0.07 c
RJ100	62.50 ± 0.64 a	19.28 ± 0.84 c	2.67 ± 0.86 a	19.47 ± 0.95 c	0.14 ± 0.04 a	37.99 ± 0.69 d

L* for lightness from black (0) to white (100), a* from green (−) to red (+), and b* from blue (−) to yellow (+), C*—chroma, *h*°—hue angle, Δ*E*—total color differences. CW—control wafers, RJ10–RJ100—wafers with 10% to 100% raspberry juice addition, respectively. All values are mean ± standard deviation for triplicate experiments. Means in the rows marked with the same letters do not differ significantly at *p* ≤ 0.05.

**Table 4:** Antioxidant properties of the control and fortified wafers.

Antioxidant Activity	Sample					
	C	RJ10	RJ20	RJ50	RJ75	RJ100
**E**						
ABTS^•+^ (µM TE/g)	0.95 ± 0.02 aA	1.32 ± 0.06 bA	1.56 ± 0.02 cA	1.57 ± 0.04 cA	1.59 ± 0.04 cA	2.08 ± 0.03 dA
DPPH (µM TE/g)	11.99 ± 0.25 aC	12.65 ± 0.30 abC	13.25 ± 0.19 bcC	13.61 ± 0.09 cC	13.78 ± 0.14 cC	13.86 ± 0.02 cC
Fe^2+^ (mg EDTA/g)	0.26 ± 0.00 aA	0.27 ± 0.00 bA	0.28 ± 0.00 cA	0.31 ± 0.00 dA	0.34 ± 0.00 eA	0.36 ± 0.00 fA
RP (µM TE/g)	3.13 ± 0.15 aB	3.83 ± 0.05 bB	4.60 ± 0.00 cB	6.20 ± 0.07 dB	7.03 ± 0.15 eB	9.17 ± 0.30 fB
**B**						
ABTS^•+^ (µM TE/g)	0.73 ± 0.18 aA	1.22 ± 0.06 bA	1.36 ± 0.07 bcA	1.78 ± 0.15 cdA	2.18 ± 0.02 deB	2.38 ± 0.07 eA
DPPH (µM TE/g)	7.38 ± 0.21 aA	7.90 ± 0.30 abA	8.62 ± 0.16 bcA	6.82 ± 0.16 abcA	8.05 ± 0.14 cA	9.16 ± 0.05 dA
Fe^2+^ (mg EDTA/g)	0.42 ± 0.00 aC	0.44 ± 0.00 bC	0.45 ± 0.00 cC	0.45 ± 0.00 cC	0.45 ± 0.00 dC	0.46 ± 0.00 dC
RP (µM TE/g)	0.86 ± 0.07 aA	1.23 ± 0.01 bA	1.55 ± 0.05 cA	1.74 ±0.07 dA	2.86 ± 0.07 eA	4.34 ± 0.02 fA
**D**						
ABTS^•+^ (µM TE/g)	11.11 ± 0.07 aB	11.41 ± 0.02 abB	11.74 ± 0.07 bcB	11.75 ± 0.17 bcB	11.99 ± 0.06 cdC	12.28 ± 0.06 dB
DPPH (µM TE/g)	9.88 ± 0.19 aB	10.39 ± 0.25 abB	10.63 ± 0.05 bcB	10.57 ± 0.19 abcB	11.19 ± 0.19 cB	11.91 ± 0.14 dB
Fe^2+^ (mg EDTA/g)	0.36 ± 0.00 aB	0.36 ± 0.00 bB	0.37 ± 0.00 cB	0.39 ± 0.00 dB	0.48 ± 0.00 eB	0.42 ± 0.00 fB
RP (µM TE/g)	10.31 ± 0.37 aC	12.30 ± 0.05 bC	14.62 ± 0.05 cC	16.18 ±0.22 dC	18.52 ± 0.30 eC	24.79 ± 0.57 fC

E—ethanolic extract; B—buffer extract; D—extract after in vitro digestion. CW—control wafers, RJ10–RJ100—wafers with raspberry juice addition (from 10% to 100% water substitution, respectively). ABTS^•+^—(2,2′-azino-bis(3-ethylbenzthiazoline-6-sulfonic acid, DPPH -di(phenyl)-(2,4,6-trinitrophenyl)iminoazanium, TE—Trolox equivalent, EDTA—EDTA equivalent. All values are mean ± standard deviation for triplicate experiments. Different capital letters indicate statistically significant differences within the same sample, but between the different types of extracts (*p* < 0.05). Values denoted by different small letters indicate statistically significant differences within the rows (*p* < 0.05).

**Table 5:** Lipoxygenase (LOX) and cyclooxygenase 1 and 2 (COX1, COX2) inhibitory activity of extracts from the control and fortified wafers.

Enzyme Inhibitory Activity IC_50_ (mg/mL)	Sample					
	CW	RJ10	RJ20	RJ50	RJ75	RJ100
E						
LOX	2.05 ± 0.03 dB	2.00 ± 0.01 dB	1.92 ± 0.02 cB	1.77 ± 0.04 bB	1.62 ± 0.05 aB	1.56 ± 0.09 aB
COX1	0.33 ± 0.02 cA	0.29 ± 0.01 bcA	0.27 ± 0.01 bA	0.26 ± 0.01 abA	0.25 ± 0.00 aA	0.25 ± 0.02 aA
COX2	-	9.87 ± 1.61 bC	8.88 ± 2.05 bC	4.93 ± 0.57 abC	4.23 ± 0.21 abC	1.48 ± 0.11 aC
B						
LOX	3.02 ± 0.05 cC	3.01 ± 0.21 bcC	2.99 ± 0.14 bcC	2.85 ± 0.11 bC	2.46 ± 0.15 aC	2.34 ± 0.09 aC
COX1	4.58 ± 1.65 cC	3.58 ± 0.92 cC	1.74 ± 0.10 bC	0.94 ± 0.15 abC	0.89 ± 0.08 aC	0.53 ± 0.02 aC
COX2	1.17 ± 0.26 cB	0.76 ± 0.06 bB	0.75 ± 0.07 bB	0.70 ± 0.01 bB	0.48 ± 0.02 abB	0.47 ± 0.00 aB
D						
LOX	0.56 ± 0.02 eA	0.52 ± 0.01 dA	0.50 ± 0.01 cdA	0.47 ± 0.02 cA	0.43 ± 0.01 bA	0.39 ± 0.01 aA
COX1	0.47 ± 0.05 bcB	0.44 ± 0.00 bcB	0.43 ± 0.05 bB	0.37 ± 0.01 abB	0.34 ± 0.02 aB	0.34 ± 0.00 aB
COX2	0.44 ± 0.02 cA	0.30 ± 0.01 bcA	0.30 ± 0.01 bA	0.29 ± 0.00 abA	0.29 ± 0.00 aA	0.29 ± 0.01 aA

E—ethanol extract; B—buffer extract; D—extract after in vitro digestion. CW—control wafers, RJ10–RJ100—wafers with raspberry juice addition (from 10% to 100% water substitution, respectively). All values are mean ± standard deviation for triplicate experiments. Different capital letters indicate statistically significant differences within the same sample, but between the different types of the extracts (*p* < 0.05). Values denoted by different small letters indicate statistically significant differences within the rows (*p* < 0.05).

## Data Availability

All relevant data are included in the article.
